# Rates of spectacle wear in early childhood in the Netherlands

**DOI:** 10.1186/s12887-022-03467-z

**Published:** 2022-07-12

**Authors:** Vasanthi Iyer, Clair A. Enthoven, Paula van Dommelen, Ashwin van Samkar, Johanna H. Groenewoud, Vincent V. W. Jaddoe, Sijmen A. Reijneveld, Caroline C. W. Klaver

**Affiliations:** 1 Department of Child Health, TNO, PO Box 3005, Leiden, 2301DA The Netherlands; 2 Department of Ophthalmology, Erasmus Medical Center, Postbus 2040, Rotterdam, 3000CA The Netherlands; 3 Department of Epidemiology, Erasmus Medical Center, Postbus 2040, Rotterdam, 3000CA The Netherlands; 4 The Generation R Study Group, Erasmus Medical Center, Postbus 2040, Rotterdam, 3000CA The Netherlands; 5 Resident in Elderly Medicine, Omring, Azalealaan 18, Lutjebroek, 1614SN The Netherlands; 6 Rotterdam University of Applied Sciences, Rotterdam, The Netherlands; 7 Department of Health Sciences, University Medical Center Groningen, University of Groningen, Postbus 30.001, Groningen, 9700RB The Netherlands; 8 Department of Ophthalmology, Radboudumc, Postbus 9101, Nijmegen, 6500HB The Netherlands; 9 Institute of Molecular and Clinical Ophthalmology, Mittlere Street 91, 4056 Basel, Switzerland

**Keywords:** Refractive error, Spectacle wear, Myopia, Well-child care, Policy

## Abstract

**Background:**

Refractive errors are relatively common all around the world. In particular, early onset myopia is associated with a significant burden in later life. Little is known about refractive errors in preschool children. The aim of this study was to assess the prevalence of spectacle wear, visual acuity and refractive errors in young Dutch children.

**Methods:**

We analyzed data of three prospective population-based studies: 99,660 3- to 5-year-olds undergoing vision screening at preventive child healthcare organizations, 6934 6-year-olds from the Generation R study, and 2974 7-year-olds from the RAMSES study. Visual acuity was measured with Landolt-C or LEA charts, spectacle wear was assessed, and refractive errors at age 6 and 7 were measured with cycloplegic refraction.

**Results:**

The prevalence of spectacle wear ranged from 1.5 to 11.8% between 3 to 7 years with no significant gender differences. Among children with spectacle wear at 6 years (*N* = 583) and 7 years (*N* = 350) 29.8 and 34.6% had myopia respectively, of which 21.1 and 21.6% combined with astigmatism; 19.6 and 6.8% had hyperopia, 37.2 and 11.1% hyperopia and astigmatism, and 12.5 and 32.7% astigmatism only.

**Conclusions:**

Spectacle wear in European children starts early in preschool and increases to a relatively frequent visual aid at school age. Advocating early detection and monitoring of refraction errors is warranted in order to prevent visual morbidities later in life.

**Supplementary Information:**

The online version contains supplementary material available at 10.1186/s12887-022-03467-z.

## Background

Refractive errors (myopia, hyperopia and astigmatism) are relatively common all around the world. They can be easily corrected using spectacles, making their early detection an important component of well-child care [1]. The prevalence of myopia in particular has increased dramatically in the last decades and is expected to affect half of the world’s population by 2050 [2]. In East Asia, 80 to 90% of the young adults is myopic; Europe and the USA are following with respectively 50 and 40% [3, 4]. Our changing lifestyles such as less outdoor exposure and higher educational levels with corresponding increase in near vision activities in childhood, such as reading, smartphone and computer use are considered risk factors for myopia development and are likely to cause the increase in development [5–7].

Babies are born with an average hyperopic refraction of + 2.00 diopters (D) following a Gaussian distribution. Their refractive development decreases towards emmetropia following a narrower leptokurtic distribution in infancy and childhood. This process is called emmetropisation and is largely completed by the age of 6 years [8]. An infant with a refraction on the left side of the distribution will more likely develop myopia later in childhood [9]. Early onset myopia increases the risk of high myopia (≤ − 6.00 D) in adulthood. High myopia consequently increases the risk of complications in the posterior segment of the eye later in life, which may result in irreversible visual impairment or even blindness [10, 11]. It is therefore important to prevent myopia development in young children by means of lifestyle changes, i.e. more outdoor light exposure and balanced near vision activities [5, 6, 12].

Pediatric vision screening programs emphasize early detection and provision of appropriate visual rehabilitation to prevent or minimize visual disability. Many high income countries have services for the early detection of vision problems in children [13]. Effective vision screening is necessary and altogether challenging in pre-school aged children, and referral for services is yet another large challenge for primary care pediatric providers [14]. In the USA, most children visit a pediatrician who provides pediatric primary care. In the Netherlands, preventive child healthcare services screen all children for general health problems and visual acuity with charts at the ages of 3, 4 and 5 to 6 years. Amblyopia, colloquially called “lazy eye,” is an important focus of vision screening programs in Europe as well as the USA [14, 15]. In this study, we assessed the prevalence of spectacle wear, visual acuity and refractive errors in three Dutch cohorts of (pre-)school children. This knowledge will provide insight to direct vision screening programs in early youth.

## Materials and methods

### Setting and population

We analyzed three population-based studies from the Netherlands: the Preventive Child Healthcare Registry (PCHR), Generation R study, and the Rotterdam Amblyopia Screening Effectiveness Study (RAMSES). PCHR is a cross-sectional study in which population vision screening was performed as part of preventive child health care by Dutch organizations. These organizations provide community-based preventive services free of charge, reaching about 95% of all children [14]. Permission was obtained from three organizations involving mostly villages and towns from the south-east to the south-west of the Netherlands to use the data collected in a national database. The database only included the age and gender of each patient at the time of screening. The study population consisted of 99,660 children born between 2008 and 2015 who participated in vision screening around the age of 3 years (36-41 months; *N* = 14,018), 4 years (42-59 months; *N* = 45,178) and/or 5 years (60-83 months; *N* = 50,140) between 2013 and 2018. Most of the children (90.8%) participated in only one vision screening (*N* = 10,711 at age 3, *N* = 36,338 at age 4 and *N* = 43,460 at age 5), some (9.0%) participated in two vision screenings (*N* = 3093 at age 3, *N* = 8626 at age 4 and *N* = 6466 at age 5), and only few children (0.2%) participated in all three vision screenings (*N* = 214 at ages 3, 4 and 5). The PCHR database is unique and has never been analyzed before.

Since the PCHR database did not contain information on the results of referral after screening, we also performed a secondary analysis of the relevant data from Generation R and RAMSES. Both studies are prospective multi-ethnic population-based cohorts from Rotterdam. Regarding Generation R, 9778 pregnant women were included in this study and children were born between 2002 and 2006. At 6 years of age, these children were invited for examination at the research center. Of the initial cohort, 6690 children participated in the physical examination (mean age 6 years, range 5 to 9 years; response rate 68.4%). The complete methodology has been described elsewhere [16]. Regarding RAMSES, children born between 1996 and 1997 were included and their vision was regularly measured as part of a Dutch screening program. Of the 4624 children at baseline, 2974 underwent a final eye examination at 7 years (mean age 7 years, range 6 to 8 years; response rate 46.3%). The complete methodology has been described elsewhere [14].

### Procedure and measures

#### Preventive Child Healthcare Registry (PCHR)

Age, gender, presenting spectacle wear and visual acuity were assessed. Spectacle wear was defined as those who already had spectacles (presenting spectacle wear). Uncorrected visual acuity was measured with Landolt C charts at a distance of five meters. Children with reduced visual acuity were referred to an ophthalmologist or orthoptist for further assessment. Visual acuity data of children who were not screened with the Landolt C charts (36.3% at 4 years, and 7.0% at 5 years) were excluded. In accordance with the Dutch vision screening guidelines, visual acuity was not assessed at the screening center in children with spectacles, this is performed at the hospital.

#### Generation R

Age, gender, spectacle wear, visual acuity and refractive errors were registered. Presenting visual acuity was measured with LEA charts at a distance of three meters [17]. Children with reduced visual acuity > 0.1 LogMAR (Logarithm of the Minimum Angle of Resolution, 0.8 decimal) were referred to the orthoptist or ophthalmologist for cycloplegic refractive error measurements. Spectacle wear (needed) was based on the examination of the orthoptist and determined by visual acuity and cycloplegic refractive error measurements after referral.

#### Rotterdam AMblyopia Screening Effectiveness Study (RAMSES)

In this study, age, gender and visual acuity with Landolt C chart were registered. Children with visual acuity of > 0.1 LogMAR (0.8 decimal) were referred to the study orthoptists for visual acuity measurement with a Snellen chart and retinoscopy under cycloplegia if deemed necessary. Children with spectacle wear and visual acuity of ≤0.1 LogMAR (0.8 decimal) during the screening were not further investigated. Spectacle wear (needed) was based on the judgement of the orthoptist who performed the visual acuity and cycloplegic refractive error measurements after referral.

### Outcome measures

The main outcome measure was spectacle wear at age 3, 4, 5, 6 and 7 years. The second outcome measure was reduced visual acuity, which is assessed at 4, 5, 6 and 7 years and classified according to three criteria: > 0.1 LogMAR (0.8 decimal) in OD (right eye) and in OS (left eye) separately, > 0.3 LogMAR (0.5 decimal) in OD and OS separately, and ≥ 2 LogMAR lines difference between OD and OS. At the age of 4 and 5 years > 0.3 LogMAR (0.5 decimal) is considered abnormal. At ages 6 and 7 years, > 0.1 LogMAR (0.8 decimal) is considered abnormal. Amblyopia is defined as an interocular acuity difference of ≥2 LogMAR lines, indicated on the chart. Refractive errors among children with spectacle wear were measured at 6 and 7 years. Spherical equivalent of refraction of the most severely affected eye was calculated in diopters (D) as sphere + ½ cylinder. The most severely affected eye was chosen, instead of the random or right eye, to investigate the main reason for spectacle wear. Myopia was defined as spherical equivalent of ≤ − 0.5 D and hyperopia as spherical equivalent ≥ + 1.0 D and astigmatism was defined as cylindrical power of ≤ − 0.75 D. Sensitivity analysis were performed with astigmatism defined as cylindrical power of ≤ − 2.00 D in order to better compare with other studies.

### Statistical analyses

The proportion of spectacle wear and reduced visual acuity were calculated by dividing the number of children with spectacle wear or reduced visual acuity by the total sample size times 100% for the ages 3, 4, 5, 6 and 7 years specifically. Differences between males and females in spectacle wear were tested using chi-square tests. The proportion of refractive error categories was calculated among 6 and 7-year-old children with spectacle wear, and for the whole study sample (% spectacle wear * % refractive error category). The distribution of spherical equivalent was plotted for both cohorts*.* All analyses were performed using SPSS Statistics program (Chicago, Illinois) version 25.

## Results

### Background characteristics

Within the PCHR database (at 3, 4 and 5 years), Generation R (at 6 years) and RAMSES (at 7 years), gender was equally represented; proportions of females were 49.4, 48.9, 48.9, 49.9, and 48.8%, respectively.

### Spectacle wear and visual acuity

The spectacle wear and visual acuity across the age groups are presented in Table 1. Presenting spectacle wear was 1.5% at 3 years, 2.3% at 4 years and 6.6% at 5 years. Prescribed spectacle wear was 8.2% at 6 years and 11.8% at 7 years. No gender differences in spectacle wear were found at 3 years (*p* = 0.38), 4 years (*p* = 0.75), 5 years (*p* = 0.64), 6 years (*p* = 0.45) and 7 years (*p* = 0.92). The proportion of children with visual acuity > 0.1 LogMAR (0.8 decimal) ranged between 76% at 4 years and 6% at 5-7 years. The proportion of children with visual acuity > 0.3 LogMAR (0.5 decimal) ranged between 11% at 4 years, and 0.4% at 6 years. The proportion of children with ≥2 LogMAR lines difference between OD and OS ranged between 3.8% at 4 years and 4.6% at 7 years.

**Table 1:** Spectacle wear and visual acuity across age groups

Source	3 years	4 years	5 years	6 years	7 years
	Youth Health Care Registry			Generation R	RAMSES
Number	**14,018**	**45,178**	**50,140**	**6690**	**4624**
Spectacle wear (%)	**1.5**	**2.3**	**6.6**	**8.2**	**11.8**
Visual acuity OD (% < 0.8, abnormal > 4 years	**–**	**76.4**^**a**^	**5.9**^**a**^	**6.0**^**b**^	**6.2**^**b**^
Visual acuity OS (% < 0.8, abnormal > 4 years)	**–**	**76.2**^**a**^	**6.0**^**a**^	**6.2**^**b**^	**6.2**^**b**^
Visual acuity OD (% < 0.5, abnormal)	**–**	**10.1**^**a**^	**1.1**^**a**^	**0.4**^**b**^	**2.2**^**b**^
Visual acuity OS (% < 0.5, abnormal)	**–**	**10.8**^**a**^	**1.2**^**a**^	**0.7**^**b**^	**2.0**^**b**^
≥2 lines difference OD and OS (%) amblyopia	**–**	**3.8**^**a**^	**2.7**^**a**^	**0.9**^**b**^	**4.6**^**b**^

^a^Presenting visual acuity without spectacles

^b^Presenting visual acuity regardless of spectacles

### Refractive errors at 6 and 7 years

**Fig. 1:**
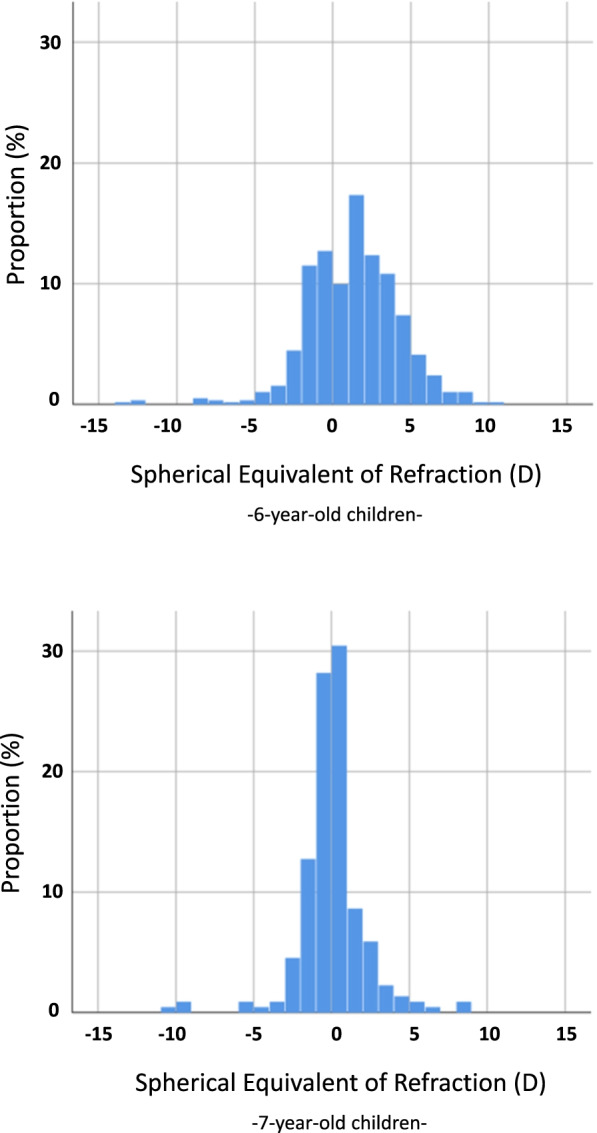
Distribution of spherical equivalent (in diopter) in children with spectacle wear at 6 (Above) and 7 years (Below)

Data on refractive errors were available in school children and are presented in Table 2. Of the children with spectacles at 6 years (*N* = 583) and 7 years (*N* = 350), information on refractive errors was available for 582 and 162 children, respectively. Of them, 29.8 and 34.6% had myopia respectively, of which 21.1 and 21.6% combined with astigmatism; 19.6 and 6.8% respectively had hyperopia, 37.2 and 11.1% hyperopia and astigmatism, and 12.5 and 32.7% astigmatism only in the most severely affected eye. When we apply these percentages to the whole study sample including those without spectacles, 2.4% of the 6-year-olds and 4.0% of the 7-year-olds had myopia with or without astigmatism. When astigmatism is defined as cylindrical power of ≤ − 2.00 D, only 0.3% of the 6-year-olds and 1.5% of the 7-year-olds had astigmatism only (Table S1). The distributions of spherical equivalent of the most severely affected eye at 6 and 7 years are shown in Fig. 1.

**Table 2:** Distribution of refractive errors in children with spectacle wear and in the whole study samples

	Generation R 6 years		RAMSES 7 years	
	N	% of the whole study sample	N	% of the whole study sample
Myopia (≤ −0.5 D)	**51**	**0.7%**	**21**	**1.5%**
Myopia (≤ −0.5 D) and astigmatism (≤ − 0.75 D)	**123**	**1.7%**	**34**	**2.5%**
Hyperopia (≥ + 1.0 D)	**114**	**1.6%**	**11**	**0.8%**
Hyperopia (≥ + 1.0 D) and astigmatism (≤ −0.75 D)	**217**	**3.1%**	**18**	**1.3%**
Astigmatism alone (≤ −0.75 D)	**73**	**1.0%**	**54**	**3.9%**
Other (emmetropia/ anisometropia)	**4**	**0.1%**	**24**	**1.7%**

## Discussion

The aim of this study was to assess the prevalence of spectacle wear, visual acuity and refractive errors in young Dutch (pre) school children. Our study among > 100,000 3- to 7-year-old children from the Netherlands showed that the prevalence of spectacle wear ranged between 1.5% at 36 months and 11.8% at 7 years. Among all children with spectacles around the age of 6 and 7 years, most children had hyperopia or astigmatism and 2.4-4.0% of the children aged 6 and 7 years had myopia.

The prevalence of spectacle wear in our study was 1.5% at 3 years and increased up to 11.8% at 7 years. In the case of the PCHR database, the prevalence of *presenting* spectacle wear was provided and in the Generation R and RAMSES databases we calculated the prevalence of spectacles *needed.* We performed a literature search on spectacle wear within the age range 0-7-year-olds published the past 10 years and found a wide range in the prevalence of *presenting* and *needed* spectacle wear between countries. The highest prevalence of *presenting* spectacle wear was 12.8% among 6- to 7-year-old children from Ireland, while the lowest prevalence was 3.4% in 4.5- to 7-year-old children from Denmark and 5- to 6-year-old children from Pakistan [18–20]. The highest prevalence of spectacles *needed* was 21.0% among 3- to 6-year-old children from Medina in western Saudi Arabia; the lowest 4.5% among 4- to 8-year-old children from Riyadh in Saudi Arabia (Fig. S1 Spectacle wear among 3- to 10-year-old children in the world; Table S2 Spectacle wear and refractive errors in other childhood population-based studies) [21, 22].

The population vision screening in the Netherlands is free of charge reaching about 95% of all children. We believe that a fair comparison regarding spectacle wear can be performed within the Netherlands, but comparisons with other countries are more difficult. In countries without extensive screening program, spectacle wear may be an underestimation of the proportion of children that actually *need* spectacles [19]. Socioeconomic factors may also play a role, with higher rates of uncorrected refractive error in less developed areas. Also, the need for spectacles may be different between countries or studies and is often based on clinical judgement. In the Netherlands, spectacles prescription depends on the degree of refractive errors measured using cycloplegia, but also accounting for age, presence of any amblyogenic factors such as anisometropia or strabismus, and for other complaints. In the study of Alrahili et al. [22], non-cycloplegic automated refractive error measurements were performed which may have led to the high percentage of spectacle prescription.

Reduced visual acuity hints towards the presence of uncorrected refractive errors. In our study, presenting spectacle wear was determined at ages 3 to 5 years, and was 1.5 and 6.6%, respectively. This proportion is likely to be close to the proportion of spectacles needed at age 5 years, because reduced visual acuity (> 0.3 LogMAR) was only 1% at this age. The proportion of spectacles needed at age 3 and 4 years, however, may have been higher because diminished visual acuity (> 0.3 LogMAR) occurred up to 10% at 4 years. Slightly reduced visual acuity > 0.1 LogMAR at 4 years was relatively frequent, which probably is due to errors because of the difficulty of testing visual acuity in children at this age [14].

Most of the 6- and 7-year-old children with spectacles in our study had astigmatism (71 and 65%), 57 and 18% had hyperopia, and 30 and 34% had myopia respectively. Studies have reported a large variance in astigmatism prevalence, e.g., 69% of the 3- to 5-year-old children from the United States wear spectacles for astigmatism compared to 40% of the 4–5-year-old children from the United Kingdom [23, 24]. When astigmatism was defined as ≤ − 2.00 D, only 23 and 12% of the 6- and 7-year-old children with spectacles had astigmatism (with or without myopia or hyperopia) in our study. With respect to spherical refractive errors, hyperopia was more common in the 6-year-old children, whereas myopia was more common in the 7-year-old children in our study. When comparing to other countries, hyperopia is more common in Europe, whereas myopia is much more common in East Asia. For example, among 4 to 7-year-old Danish children with spectacle wear, 71% had hyperopia (> 3.5 diopter) and none of them had myopia, while among 6-year-old Chinese children only 9% had hyperopia (≥2 diopter) and 45% already had myopia (≤ − 0.5 diopter) [19, 25]. During the process of visual development over time, refractive error changes from hyperopia to emmetropia. Newly prescribed spectacles at older ages are therefore more likely spectacles to correct for myopia.

A large study on European populations showed a general shift towards myopia over generations, an increase in myopia prevalence and a decrease in hyperopia prevalence [3]. The results of our study show a myopia prevalence of 2.4 and 4.0% in 6- and 7-year-old children, respectively. An early age of onset of myopia is associated with high myopia (≤ − 6.00 D) in adulthood. In turn, high myopia in adulthood is associated with complications that may lead to visual impairment later in life [10, 11]. Prevention of early onset myopia may therefore indirectly prevent visual loss due to myopia later in life. Since lifestyle in childhood is a prominent risk factor, precautionary actions by well-child care professionals to stimulate outdoor exposure and limit screen time are warranted [5, 6, 12]. Besides aiming for prevention of amblyopia, vision screening in young children should also focus on refractive errors, and in particular on myopia.

## Strengths and limitations

Our study had several strengths and limitations. Strengths are the large number of children screened over a substantial region of the Netherlands resulting in a unique database, and the uniform guideline-based vision screening for different age-groups in the PCHR study.

A limitation is the number of missing data on refractive error of the children with spectacles in the RAMSES study. This may have led to an overestimation of myopia, because hyperopia remains relatively stable whereas myopia usually increases in childhood. In our analysis we assume the missing refractive error is non-differential. Another limitation is the relatively large number of missing data for visual acuity at 4 years, because we only included visual acuity measurements with the validated Landolt C chart. Whether or not Landolt C charts were used mainly depended on organizational choices, this is therefore unlikely to have resulted in a systematic under- or overrepresentation of visual acuity. Third, when children are referred to services, recommended spectacles are not always bought or if bought not used. We therefore collected refractive error data from the prescriptions of the orthoptist or ophthalmologist to ensure we had complete measurements, thus preventing potential bias.

## Conclusion

Spectacles have become a relatively frequent visual aid in young children which already increases in preschool years. A gradual increase of visual aid with age indicates a need to monitor these changes including behavioral changes like the early use of mobile devices. Advocating early detection of refraction errors is warranted. Effective vision screening and follow-up after referral for services are points of concern. As early-onset myopia has major consequences for visual acuity later in life, awareness and advice for preventive behavior by professionals in well-child care will improve the visual prognosis, and thus highly improve child health.

## Supplementary Information

**Additional file 1: Figure S1.** Spectacle wear among 3-10-year-old children in the world.

**Additional file 2: Table S1.** Distribution of refractive errors in children with spectacle wear and share of the full study samples.

**Additional file 3: Table S2.** Spectacle wear and refractive errors in other childhood population-based studies.

## Data Availability

The datasets generated and/or analyzed during the current study are not publicly available because participants were assured raw data would remain confidential and would not be shared with the public. Due to the sensitivity of the data and the restrictions from the informed consent, the data will not be stored at a public repository, but are available from the corresponding author on reasonable request.

## Abbreviations

PCHR: Preventive Child Healthcare Registry
Generation R: Generation Rotterdam
RAMSES: Rotterdam AMblyopia Screening Effectiveness Study
OD: Right eye
OS: Left eye
LogMar: Logarithm of the Minimum Angle of Resolution
