# Supplementary material for: Rates of spectacle wear in early childhood in the Netherlands
Source: BMC Pediatr. 2022 Jul 12;22:409. doi: 10.1186/s12887-022-03467-z (PMC9275042; doi:10.1186/s12887-022-03467-z)
Supplement: Supplementary file 1 — Additional file 1: Figure S1. Spectacle wear among 3-10-year-old children in the world. [file 12887_2022_3467_MOESM1_ESM.docx]

**
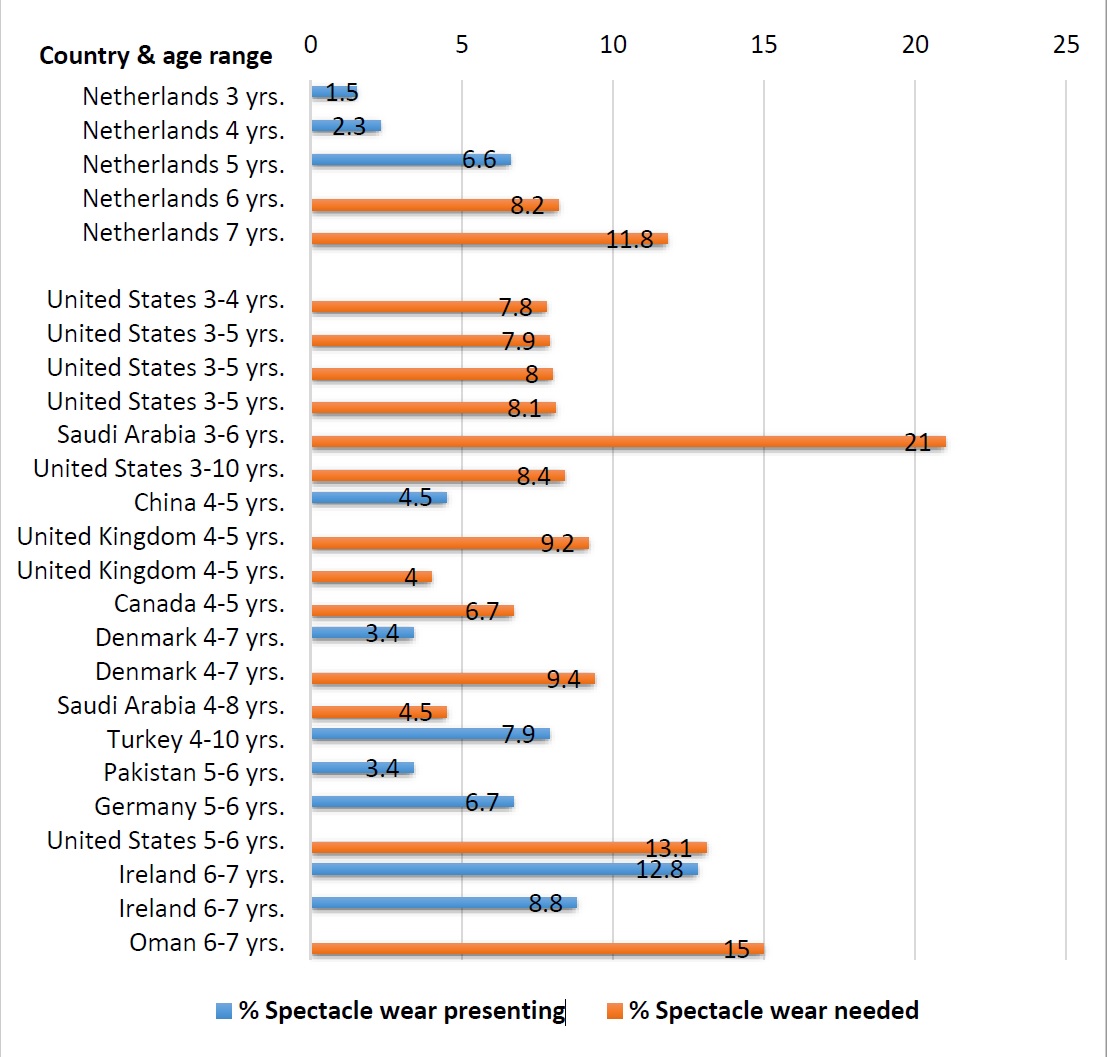
**

**Country & age range**

Netherlands 3 yrs.

Netherlands 4 yrs.

Netherlands 5 yrs.

Netherlands 6 yrs.

Netherlands 7 yrs.

United States 3-4 yrs.

United States 3-5 yrs.

United States 3-5 yrs.

United States 3-5 yrs.

Saudi Arabia 3-6 yrs.

United States 3-10 yrs.

China 4-5 yrs.

United Kingdom 4-5 yrs.

United Kingdom 4-5 yrs.

Canada 4-5 yrs.

Denmark 4-7 yrs.

Denmark 4-7 yrs.

Saudi Arabia 4-8 years

Turkey 4-10 yrs.

Pakistan 5-6 yrs.

Germany 5-6 yrs.

United States 5-6 yrs.

Ireland 6-7 yrs.

Ireland 6-7 yrs.

Oman 6-7 yrs.

**% Spectacle wear presenting** % **Spectacle wear needed**
