# Supplementary material for: Rates of spectacle wear in early childhood in the Netherlands
Source: BMC Pediatr. 2022 Jul 12;22:409. doi: 10.1186/s12887-022-03467-z (PMC9275042; doi:10.1186/s12887-022-03467-z)
Supplement: Supplementary file 2 — Additional file 2: Table S1. Distribution of refractive errors in children with spectacle wear and share of the full study samples. [file 12887_2022_3467_MOESM2_ESM.docx]

**Table S1: Distribution of refractive errors in children with spectacle wear and share of the full study samples**

|  | Generation R 6 years | | RAMSES 7 years | |
| --- | --- | --- | --- | --- |
|  | N | % of the full study sample | N | % of the full study sample |
| Myopia (≤ -0.5 D) | **124** | **1.7%** | **42** | **3.1%** |
| Myopia (≤ -0.5 D) and astigmatism (≤ -2.00 D) | **50** | **0.7%** | **13** | **0.9%** |
| Hyperopia (≥+1.0 D) | **265** | **3.7%** | **21** | **1.5%** |
| Hyperopia (≥+1.0 D) and astigmatism (≤ -2.00 D) | **66** | **0.9%** | **8** | **0.6%** |
| Astigmatism alone (≤ -2.00D) | **20** | **0.3%** | **20** | **1.5%** |
| Other (emmetropia/ anisometropia) | **57** | **0.8%** | **58** | **4.2%** |
