# Supplementary material for: Rates of spectacle wear in early childhood in the Netherlands
Source: BMC Pediatr. 2022 Jul 12;22:409. doi: 10.1186/s12887-022-03467-z (PMC9275042; doi:10.1186/s12887-022-03467-z)
Supplement: Supplementary file 3 — Additional file 3: Table S2. Spectacle wear and refractive errors in other childhood population-based studies. [file 12887_2022_3467_MOESM3_ESM.docx]

**Table S2 Spectacle wear and refractive errors in other childhood population-based studies.**

| Authors (year) | Number of participants | Data collection (year) | Country | Age (years) | Ethnicity | Male (%) | Spectacle wear (%) |
| --- | --- | --- | --- | --- | --- | --- | --- |
| Hu et al., 2012 (1) | 2830 | 2006-2007 | United States | 3-4 | - | - | 7.8 (needed) |
| Margines et al., 2020 (2) | 79,451 | 2012-2017 | United States | 3-5 | 79% Latino | 51 | 7.9 (needed) |
| Hendler et al., 2016 (3) | 11,260 | 2012-2013 | United States | 3-5 | Hispanic | - | 8.0 (needed) |
| Mehravaran, et al., 2016 (4) | 12,088 | 2012-2013 | United States | 3-5 | 86% Latino | 50.8 | 8.1 (needed) |
| Alrahili et al., 2017 (5) | 865 | 2015 | Saudi Arabia | 3-6 | - |  | 21.0 (needed) |
| Griffith et al., 2016 (6) | 63,841 | 2002-2014 | United States | 3-10 | - | 49.4 | 8.4 (needed) |
| Zhang et al., 2018 (7) | 1986 | 2016 | China | 4-5 | Chinese | 54.8 | 4.5 (presenting) |
| Toufeeq and Oram, 2014 (8) | 4171 | 2009-2010 | United Kingdom | 4-5 | - | - | 9.2 (needed) |
| Garretty et al., 2017 (9) | 7807 | 2013-2014 | United Kingdom | 4-5 | - | - | 4.0 (needed) |
| Nishimura et al., 2020 (10) | 4811 | 2015-2017 | Canada | 4-5 | - | - | 6.7 (needed) |
| Sandfeld et al., 2019 (11) | 447 | 2015-2016 | Denmark | 4.5-7 | 87% White | 52.5 | 3.4 (presenting)  9.4 (needed) |
| Al-Rowaily., 2010 (12) | 1319 | 2008 | Saudi Arabia | 4-8 | Saudi | 44 | 4.5 (needed) |
| Azizoglu et al., 2017 (13) | 823 | 2009 | Turkey | 4-10 | Turkish | 50.4 | 7.9 (presenting) |
| Ertekin et al., 2016 (14) | 476 | 2013 | Pakistan | 5-6 | Pakistani | 49.4 | 3.4 (presenting) |
| Elflein et al., 2020 (15) | 160,122 | 2010-2015 | Germany | 5.8 | White | 52.1 | 6.7 (presenting) |
| Hark et al., 2018. (16) | 275 | 2014-2016 | United States | 5-6 | - | - | 13.1 (needed) |
| O’Donoghue et al., 2010 (17) | 392 | 2006-2008 | Ireland | 6-7 | White | 49.5 | 12.8 (presenting) |
| Harrington et al., 2019 (18) | 728 | 2016-2018 | Ireland | 6-7 | 79.9% White | 51.8 | 8.8 (presenting) |
| Al Harby et al., 2016 (19) | 286 | 2012 | Oman | 6-7 | - | 35 | 15.0 (needed) |

**Table S2 References**

1. Hu VH, Starling A, Baynham SN, Wager H, Shun-Shin GA. Accuracy of referrals from an orthoptic vision screening program for 3- to 4-year-old preschool children. Journal of American Association for Pediatric Ophthalmology and Strabismus. 2012;16(1):49-52.

2. Margines JB, Huang C, Young A, Mehravaran S, Yu F, Mondino BJ, et al. Refractive Errors and Amblyopia Among Children Screened by the UCLA Preschool Vision Program in Los Angeles County. American Journal of Ophthalmology. 2020;210:78-85.

3. Hendler K, Mehravaran S, Lu X, Brown SI, Mondino BJ, Coleman AL. Refractive Errors and Amblyopia in the UCLA Preschool Vision Program; First Year Results. American Journal of Ophthalmology. 2016;172:80-6.

4. Mehravaran S, Duarte PB, Brown SI, Mondino BJ, Hendler K, Coleman AL. The UCLA preschool vision program, 2012-2013. Journal of American Association for Pediatric Ophthalmology and Strabismus. 2016;20(1):63-7.

5. Alrahili NHR, Jadidy ES, Alahmadi BSH, Abdula'al MF, Jadidy AS, Alhusaini AA, et al. Prevalence of uncorrected refractive errors among children aged 3-10 years in western Saudi Arabia. Saudi Med J. 2017;38(8):804-10.

6. Griffith JF, Wilson R, Cimino HC, Patthoff M, Martin DF, Traboulsi EI. The Use of a Mobile Van for School Vision Screening: Results of 63 841 Evaluations. American Journal of Ophthalmology. 2016;163:108-14.e1.

7. Zhang X, Wang Y, Huang D, Sun Q, Zhao X, Ding H, et al. Prevalence of reduced visual acuity among preschool children in eastern China and comparison at a 5-year interval. Clinical & Experimental Ophthalmology. 2018;46(9):994-1001.

8. Toufeeq A, Oram AJ. School-entry Vision Screening in the United Kingdom: Practical Aspects and Outcomes. Ophthalmic Epidemiology. 2014;21(4):210-6.

9. Garretty T. Final Visual Outcomes and Treatment Received for Children Referred from a UK Primary School Visual Screening Program: A Comparison of An Orthoptic-led Program with Orthoptic-delivered Services. Strabismus. 2017;25(4):184-90.

10. Nishimura M, Wong A, Dimaras H, Maurer D. Feasibility of a school-based vision screening program to detect undiagnosed visual problems in kindergarten children in Ontario. Canadian Medical Association Journal. 2020;192(29):E822.

11. Sandfeld L, Weihrauch H, Tubæk G. Analysis of the current preschool vision screening in Denmark. Acta Ophthalmologica. 2019;97(5):473-7.

12. Al-Rowaily MA. Prevalence of refractive errors among pre-school children at King Abdulaziz Medical City, Riyadh, Saudi Arabia. Saudi Journal of Ophthalmology. 2010;24(2):45-8.

13. Azizoğlu S, Crewther SG, Şerefhan F, Barutchu A, Göker S, Junghans BM. Evidence for the need for vision screening of school children in Turkey. BMC Ophthalmology. 2017;17(1):230.

14. Ertekin YH, Tekin M, Uludag A, Arikan S, Sahin EM. Vision screening in children: Is 7-9 years of age a threshold for visual impairment? Pak J Med Sci. 2016;32(5):1194-8.

15. Elflein HM, Pokora R, Müller DF, Jahn K, Ponto KA, Pitz S, et al. No Benefit of a Pediatric Screening in Discovering Reduced Visual Acuity in Children: Experiences from a Cross-Sectional Study in Germany. International Journal of Environmental Research and Public Health. 2020;17(10):3419.

16. Hark LA, Shiuey E, Yu M, Tran E, Mayro EL, Zhan T, et al. Efficacy and outcomes of a summer-based pediatric vision screening program. Journal of American Association for Pediatric Ophthalmology and Strabismus. 2018;22(4):309.e1-.e7.

17. O'Donoghue L, McClelland JF, Logan NS, Rudnicka AR, Owen CG, Saunders KJ. Refractive error and visual impairment in school children in Northern Ireland. British Journal of Ophthalmology. 2010;94:1155-9.

18. Harrington SC, Stack J, Saunders K, O’Dwyer V. Refractive error and visual impairment in Ireland schoolchildren. British Journal of Ophthalmology. 2019;103(8):1112.

19. Al Harby S, Al-Asbali T, Khandekar R. Visual acuity and refractive status of Omani students with refractive error in grades 1, 4 and 7: A retrospective cohort study. Oman journal of ophthalmology. 2016;9(1):27.
